# Haplotype-resolved chromosomal-level genome assembly of Buzhaye (***Microcos paniculata***)

**DOI:** 10.1038/s41597-023-02821-9

**Published:** 2023-12-15

**Authors:** Detuan Liu, Xiaoling Tian, Shicheng Shao, Yongpeng Ma, Rengang Zhang

**Affiliations:** 1 Yunnan Key Laboratory for Integrative Conservation of Plant Species with Extremely Small Populations, Kunming Institute of Botany, Chinese Academy of Sciences, Kunming, 650201 China; 2 CAS Key Laboratory for Plant Diversity and Biogeography of East Asia, Kunming Institute of Botany, Chinese Academy of Sciences, Kunming, 650201 China; 3 University of Chinese Academy of Sciences, Beijing, 101408 China; 4 Institute of International Rivers and Eco-Security, Yunnan University, Kunming, 650500 China; 5 CAS Key Laboratory of Tropical Forest Ecology, Xishuangbanna Tropical Botanical Garden, Chinese Academy of Sciences, Mengla, 666303 China

**Keywords:** Plant evolution, Chromosomes

## Abstract

*Microcos paniculata* is a shrub used traditionally as folk medicine and to make herbal teas. Previous research into this species has mainly focused on its chemical composition and medicinal value. However, the lack of a reference genome limits the study of the molecular mechanisms of active compounds in this species. Here, we assembled a haplotype-resolved chromosome-level genome of *M. paniculata* based on PacBio HiFi and Hi-C data. The assembly contains two haploid genomes with sizes 399.43 Mb and 393.10 Mb, with contig N50 lengths of 43.44 Mb and 30.17 Mb, respectively. About 99.93% of the assembled sequences could be anchored to 18 pseudo-chromosomes. Additionally, a total of 482 Mb repeat sequences were identified, accounting for 60.76% of the genome. A total of 49,439 protein-coding genes were identified, of which 48,979 (99%) were functionally annotated. This haplotype-resolved chromosome-level assembly and annotation of *M. paniculata* will serve as a valuable resource for investigating the biosynthesis and genetic basis of active compounds in this species, as well as advancing evolutionary phylogenomic studies in Malvales.

## Background & Summary

**Fig. 1:**
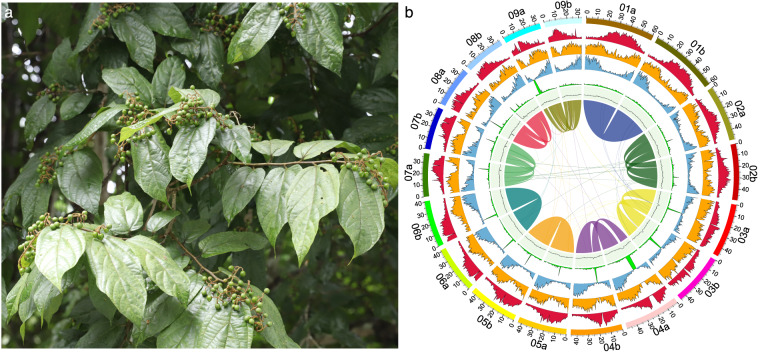
Morphological characters (**a**) and the landscape of genome assembly and annotation of *M. paniculata* (**b**). The tracks from outside to inside are: pseudo-chromosomes, density of class I TEs, density of class II TEs, density of protein-coding genes, proportion of tandem repeats, GC content and collinear blocks.

*Microcos paniculata* Linnaeus (Fig. 1a), known in Chinese as Buzhaye, is a shrub commonly used in traditional Chinese medicine and herbal cooling teas^1^, including Wanglaoji, Huoqizheng^2^ and Jiaduobao, with an annual demand of about 250 tons (http://bk.cnpharm.com/zgyyb/2008/04/28/246974.html). The leaves of *M. paniculata* are also commonly used in ethnomedicinal treatments for food stagnation, damp-heat jaundice and fever^3^. Up to now, numerous studies have extensively investigated the phytochemical composition and pharmacological properties of this species, revealing the existence of bioactive secondary metabolites such as flavonoids, alkaloids, triterpenoids and organic acids^1,4^ from *M. paniculata* extracts. However, due to the lack of a high-quality reference genome, the molecular basis and evolution of the secondary metabolite biosynthesis in *M. paniculata* are rarely reported^5^.

**Fig. 2:**
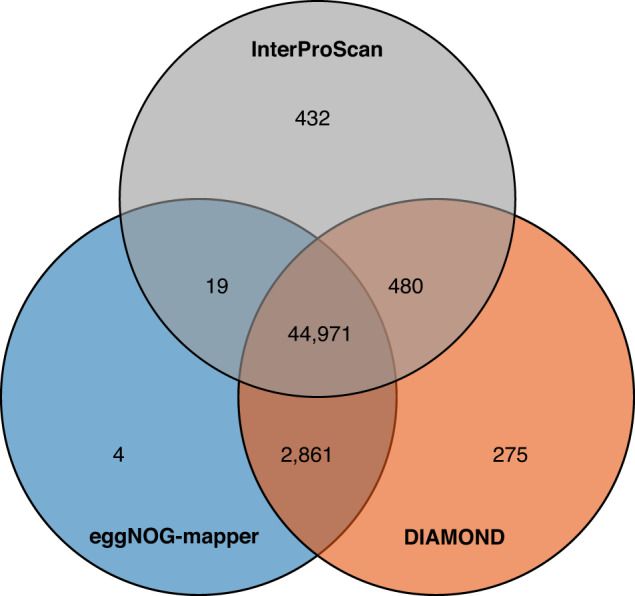
Venn diagram showing the unique and shared functionally annotated protein-coding genes in *M. paniculata* using the three strategies.

In the present study, we assembled the genome of *M. paniculata* using 106 × short reads (42 Gb), 35 × HiFi reads (14 Gb), 75 × Hi-C reads (30 Gb) and 50 × iso-seq reads (20 Gb). The final assembly (~792 Mb) consisted of two complete haplotypes, haplotype A (399.43 Mb) and haplotype B (393.10 Mb), with contig N50 lengths of 43.44 Mb and 30.17 Mb, respectively (Table 1). About 99.93% of the assembled sequences were anchored onto 18 (2n) pseudo-chromosomes (Fig. 1b). The chloroplast and mitochondrial genomes were 159,456 bp and 380,905 bp, respectively. A total of 1,080,648 repeat sequences, with an approximate length of 482 Mb were identified, accounting for 60.76% of the assembled genome. Of the identified repeats, long terminal repeats (LTRs) constituted the largest proportion, with a number of 394,112 and a cumulative length of 321,160,287 bp, accounting for 40.52% of the *M. paniculata* genome assembly (Table 2). The genome contained 65,874 genes, including 49,439 protein-coding genes and 16,435 non-coding genes (Table 3). A total of 48,979 genes were functionally annotated, accounting for 99% of the identified protein-coding genes (Table 4). Of these, 44,971 genes were annotated by all three methods together (Fig. 2). In particular, 639 genes have been annotated as being related to the biosynthesis or metabolism of flavonoids, alkaloids and triterpenoids (Table S1). The resulting high-quality reference genome and annotation of *M. paniculata* will be a valuable resource for improving our understanding of the evolutionary relationships within the Malvales, for studying the molecular basis and biosynthetic mechanisms of phytochemical compounds, and for further study and exploitation of *M. paniculata*.

**Table 1:** Summary of *M. paniculata* genome assembly.

Parameter	Genome	Haplotype A	Haplotype B
Genome size	792,535,851 bp	399,432,223 bp	393,103,628 bp
GC content	35.74%	35.73%	35.75%
Contig number	37	18	19
Contig N10	49,527,071 bp	55,167,130 bp	49,527,071 bp
Contig N50	41,049,410 bp	43,438,762 bp	30,170,985 bp
Contig N90	12,203,702 bp	13,880,047 bp	12,203,702 bp
Scaffold number	20	11	9
Scaffold N10	60,658,723 bp	60,706,172 bp	60,658,723 bp
Scaffold N50	45,573,016 bp	47,575,556 bp	45,573,016 bp
Scaffold N90	35,541,173 bp	35,541,173 bp	36,361,311 bp
Gap number	17	7	10

**Table 2:** Summary of repeat elements.

Type	Number	Length (bp)	Percent (%)	Mean length (bp)
LTRs	394,112	321,160,287	40.52	815
LINE	5,466	3,375,940	0.43	618
Helitron	154,911	42,417,336	5.35	274
TIR	188,121	59,996,054	7.57	319
Unclassified	132,568	45,978,909	5.8	347
Simple repeats	172,726	7,029,166	0.89	41
Low complexity	32,712	1,583,109	0.2	48
Polinton	32	5,983	0	187
Total	1,080,648	481,546,784	60.76	446

**Table 3:** Summary of *M. paniculata* genome annotations.

Feature	Total	Haplotype A	Haplotype B
gene	65,874	37,351	28,523
transcript	76,776	42,840	33,936
CDS	60,341	30,283	30,058
exon	363,716	187,057	176,659
intron	286,940	144,217	142,723
mRNA	49,439	24,794	24,645
rRNA	14,488	11,547	2,941
tRNA	911	478	433
other ncRNA	1,036	532	504

**Table 4:** Functional annotation of protein-coding genes in *M. paniculata*.

Program	Database	Number	Percent (%)
eggNOG-mapper	GO	22,963	46.45
	KEGG_KO	22,373	45.25
	EC	10,045	20.32
	KEGG_Pathway	14,133	28.59
	eggNOG	44,508	90.03
	COG	47,855	96.80
DIAMOND	Swiss-Prot	36,400	73.63
	TrEMBL	48,572	98.25
	NR	48,206	97.51
	TAIR10	43,580	88.15
InterProScan	CDD	16,560	33.50
	Interpro	42,031	85.02
	Gene3D	34,296	69.37
	PRINTS	7,479	15.13
	Pfam	39,734	80.37
	SMART	15,177	30.70

## Methods

### Sample collection and genome sequencing

Samples of *M. paniculata* were collected at Xishuangbanna Tropical Botanical Garden (XTBG), Chinese Academy of Sciences, Mengla, Yunnan Province, China. Genomic DNA was extracted using a modified CTAB method^6^. DNA quality was assessed using a NanoDrop One spectrophotometer (NanoDrop Technologies, Wilmington, DE, USA) and a Qubit 3.0 Fluorometer (Life Technologies, Carlsbad, CA, USA). Whole genome sequencing, Pacbio sequencing, Hi-C (high-through chromosome conformation capture) sequencing and full-length isoform sequencing (iso-seq) were performed at Wuhan Benagen Technology Co. Ltd. (Wuhan, China).

For whole genome sequencing, 1 μg of genomic DNA was sonicated to an approximate size range of 200–400 bp using a sonicator (Covaris, Brighton, UK). The short-read libraries were constructed following the manufacturer’s instructions and then sequenced on the DNBSEQ-T7 platform (BGI lnc., Shenzhen, China) using the PE (paired-end) 150 model.

For long-read sequencing, genomic DNA was sheared using the Megaruptor 3 shearing kit (Diagenode SA., Seraing, Belgium). The AMPure PB beads size selection kit (Pacbio, Menlo Park, CA, USA) was used to selectively deplete DNA fragments smaller than 5 kb. The libraries were prepared using the SMRTbell® prep kit 3.0 (Pacbio, Menlo Park, CA, USA) and then sequenced on a Revio system (Pacbio, Menlo Park, CA, USA). Raw sequencing data were converted to HiFi (high fidelity) reads using the CCS workflow 7.0.0^7^ with parameters (--streamed --log-level INFO --stderr-json-log --kestrel-files-layout–min-rq 0.9 --non-hifi-prefix fail --knrt-ada --pbdc-model).

For Hi-C sequencing, leaf material from young shoots was fixed in 2% formaldehyde solution, and the Hi-C library was generated following a published protocol^8^. Briefly, the cross-linked materials were digested with 400 units of *Mbo*I, and marked with biotin-14-dCTP, and then subjected to blunt-end ligation of crosslinked fragments. After re-ligation, reverse crosslinking and purification, the chromatin DNA was sheared to a size of 200–600 bp using sonication. The biotin-labelled Hi-C fragments were then enriched using streptavidin magnetic beads. After the addition of A-tailing and an adapter, the Hi-C libraries were PCR-amplified (12–14 cycles) and then sequenced on the DNBSEQ-T7 platform (BGI lnc., Shenzhen, China) in PE150 mode.

Full-length isoform sequencing (iso-seq) was used to obtain high quality transcriptomic data. RNA was extracted from leaves, flowers and stems of *M. paniculata* using the R6827 Plant RNA Kit (Omega Bio-Tek, Norcross, GA, USA) following the manufacturer’s instructions. The cDNA-PCR Sequencing kit SQK-PCS109 by Oxford Nanopore (Oxford Nanopore Technologies, Oxford, UK) was used to prepare full-length cDNA libraries. The libraries were then sequenced on the PromethION sequencer (Oxford Nanopore Technologies, Oxford, UK).

### Genome assembly

PacBio HiFi reads and Hi-C short reads were combined as input to Hifiasm v0.19.5-r592^9^ using the default parameters to generate haplotype-resolved contigs for subsequent analysis. Hi-C reads were mapped to the assembled haplotype contigs using Juicer v1.5.6^10^, and a Hi-C-assisted initial chromosome assembly was then performed using the 3D-DNA v180922^11^ pipeline (with the parameters --early-exit -m haploid -r 0). Chromosome boundaries were then adjusted and the misjoins and switch errors were corrected manually using Juicebox v1.11.08^12^. This process generated chromosome-scale scaffolds and un-anchored contig sequences.

**Fig. 3:**
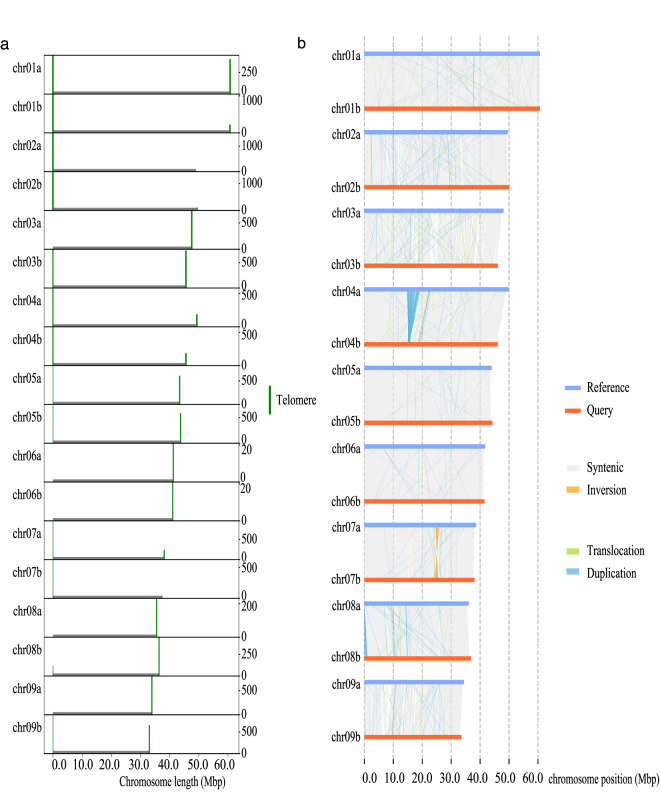
Telomere distribution (**a**) and comparation of genome structure between haplotype A and haplotype B (**b**).

LR_Gapcloser v1.1.1^13^ was used to fill gaps in the chromosome assembly based on HiFi reads (with the parameters -s p -r 2 -g 500 -v 500 -a 0.25). HiFi reads were then re-mapped to the chromosome scaffolds. The mapped reads located around the telomere repeat sequences (TTTAGGG)_n_^14^ were then extracted and assembled into contigs using Hifiasm v0.19.5-r592 with the default parameters. The resulting contigs were aligned back to the chromosome scaffold to extend the chromosome ends for telomere sequences, and totally 28 telomere sequences were obtained (Fig. 3a). In addition, GetOrganelle v1.7.5^15^ was used to assemble the chloroplast and mitochondrial genomes.

Nextpolish2 v0.1.0^16^ was used to polish the above assembly based on HiFi reads and short reads with default parameters. Redundant haplotigs and rDNA fragments were removed using the Redundans v0.13c^17^ pipeline (with the parameters -identity 0.98 -overlap 0.8) and manually curated. A high quality haplotype resolved genome assembly of *M. paniculata* was then obtained.

### Repeat annotation

The EDTA (Extensive *de novo* TE Annotator) program v1.9.9^18^ (with the parameters --sensitive 1 --anno 1) was used for the *de novo* identification of transposable elements (TE), generating a TE library. RepeatMasker v4.0.7^19^ was utilized to identify repeat elements (with the parameters -no_is -xsmall).

### Annotation of protein-coding genes and noncoding RNAs

A total of 314,962 publicly available non-redundant protein sequences from *Theobroma cacao*^20^*, Durio zibethinus*^21^*, Corchorus capsularis*^22^*, Gossypium raimondii*^23^*, Heritiera littoralis*^24^*, Dipterocarpus turbinatus*^25^*, Aquilaria sinensis*^26^*, Arabidopsis thaliana*^27^*, Carica papaya*^28^*, Vitis vinifera*^29^, and *Bombax ceiba*^30^ were used as homologous protein evidence for gene annotation. Iso-seq data were mapped to the genome using Minimap2 v2.24^31^ (with the parameters -a -x splice --end-seed-pen = 60 --G 200k), then assembled in StringTie v1.3.5^32^ (with the parameters -L -t -f 0.05), and the resulting sequences were used as transcript evidence.

PASA (Program to Assemble Spliced Alignments) v2.4.1^33^ was used to annotate the genomic structure based on transcript evidence with the default parameters. Then, full-length gene sequences were identified by aligning with homologous protein evidence using BLAT^34^ (-prot) and removing the hits with query or target coverage <95%. The gene model was trained and optimized for five rounds in AUGUSTUS v3.4.0^35^ using the full-length gene set with the default parameters.

The MAKER2 v2.31.9^36^ pipeline was used to perform annotation based on *ab initio* prediction, the transcript evidence and the homologous protein evidence. Briefly: (1) RepeatMasker v4.0.7^19^ was used to mask repeat sequences in the genome; (2) AUGUSTUS v3.4.0^35^ was used for *ab initio* prediction based on the genomic sequence; (3) BLASTN was used to align the transcript evidence to the repeat-masked genome, and BLASTX was employed to align the homologous protein evidence to the genome. Exonerate v2.2.0^37^ was used to realign the BLAST hits to the genome; (4) Finally, the predicted gene models were integrated using MAKER2 based on the hints generated from the above alignments.

EvidenceModeler (EVM) v1.1.1^38^ was further employed to merge the annotation results obtained from PASA v2.4.1 and MAKER2 v2.31.9, generating consensus annotations. TEsorter v1.4.1^39^ was utilized to identify TE protein domains on the genome (with the parameters -genome -db rexdb -cov 30 -eval 1e-5 -prob 0.9), and these domains were masked in the EVM process. The results obtained from EVM were refined by incorporating UTR sequences and alternative splicing using PASA v2.4.1 with the default parameters. Annotations that were too short (<50 amino acids), lacked start or stop codons, contained an internal stop codon, or had ambiguous bases were excluded. All annotations were then merged, and redundant annotations were removed.

In addition, for non-coding RNA (ncRNA) annotations, tRNAScan-SE v1.3.1^40^ was used to identify transfer RNA (tRNA), and Barrnap v0.9 (https://github.com/tseemann/barrnap) was used to identify ribosomal RNA (rRNA). To ensure accuracy, partial rRNA annotations were excluded. Furthermore, RfamScan v14.2^41^ was used to identify other ncRNA.

We employed three strategies to predict the function of the protein-coding genes: (1) eggNOG-mapper v2.0.0^42^ (--target_taxa Viridiplantae -m diamond) was utilized to search for homologous genes in the eggNOG database, enabling Gene Ontology (GO) and Kyoto Encyclopedia of Genes and Genomes (KEGG) annotation; (2) DIAMOND v0.9.24^43^ (--evalue 1e-5 --max-target-seqs  5) was employed to align protein-coding genes with the Swiss-Prot, TrEMBL, NR (non-redundant protein in NCBI), and the TAIR10 protein databases; (3) InterProScan v5.27-66.0^44^ was used to annotate protein domains and motifs by searching multiple publicly available databases, such as PRINTS, Pfam, SMART, PANTHER, and CDD of the InterPro database. TBtools v1.132^45^ was then used to draw a Venn diagram to show unique and shared protein-coding genes annotated using the three described strategies.

### Comparison between haplotype assemblies

SyRI (Synteny and Rearrangement Identifier) v1.6^46^ was used to detect synteny and genomic structural variations (≥50 bp in size) between the two haplotypes, with the default parameters. In total, our analysis identified 3,011 syntenic regions (∼350 Mb), 768 translocations (∼45 Mb), 20 inversions (∼2 Mb), 2,175 duplications in haplotype A (~15 Mb) and 1,686 duplications in haplotype B (~8 Mb). Most duplications were found on chromosomes 4 and 8, and most inversions were found on chromosome 7 (Fig. 3b). SyRI v1.6 was also used to identify SNPs, small InDels (insertions and deletions, <50 bp in size) and tandem repeats. Finally, 1,264,264 SNPs (∼1 Mb), 105,563 insertions (∼2 Mb in haplotype B), 100,073 deletions (∼2 Mb in haplotype A) and 282 tandem repeats (∼1 Mb) were identified.

## Data Records

The BGI short reads, PacBio HiFi long reads, Hi-C reads and Iso-Seq data have been deposited at the Sequence Read Archive database of NCBI (National Center for Bioinformation Information) under accession numbers SRR25456891-SRR25456894^47–50^. The final genome assembly has been deposited at the GenBank database under the accession numbers GCA_030664735.1^51^ and GCA_030664755.1^52^. The genome annotations are available from the Figshare repository^53^. The AUGUSTUS model trained and optimized for this genome, together with the configuration files for MAKER are available from the Figshare repository^54^.

## Technical Validation

We first calculated the mapping rate as a measure of assembly accuracy. The short reads and the long reads were re-mapped to the assembly using BWA-MEM v0.7.17-r1188^55^ and Minimap2 v2.24^31^, respectively, with the default parameters. The mapping rates were calculated after filtering out non-primary alignments. In total, 99.89% of HiFi reads, 97.75% of iso-seq reads and 99.81% of short reads were mapped (Table 5). Moreover, the read coverage depth of both short and long read data was evenly distributed along each phased chromosome, indicating high quality of our haplotype-resolved assembly (Figure S1).

**Table 5:** Summary of mapping rates.

Data set	Reads mapped	Bases mapped	≥1×	≥5×	≥10×	≥20×
HiFi reads	99.89%	99.88%	99.99%	99.79%	96.46%	32.19%
Iso-Seq reads	97.75%	99.13%	20.77%	11.37%	8.59%	6.34%
Short reads	99.81%	99.81%	99.97%	99.89%	99.73%	98.52%

**Fig. 4:**
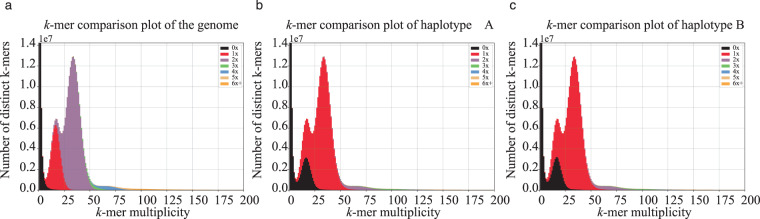
Copy number spectra plots for genome (**a**), haplotype A (**b**) and haplotype B (**c**) using KAT (K-mer Analysis Toolkit). The *k*-mers from HiFi reads display two dominant heterozygous (multiplicity = 18) and homozygous (multiplicity = 34) peaks, and those from assemblies have 0–6×+ copy numbers.

We evaluated the completeness of the genome assembly using BUSCO (Benchmarking Universal Single-Copy Orthologs) v5.3.2^56^ based on the embryophyta_odb10 ortholog database. The BUSCO evaluation of the haplotype A identified 1,591 complete BUSCOs (including 1,561 single and 30 duplicated BUSCOs), accounting for 98.6% of the haplotype, while the missing BUSCOs represented merely 0.7% (Table 6). Similarly, the BUSCO assessment of the haplotype B identified 1,588 complete BUSCOs (including 1,560 single and 28 duplicated BUSCOs), accounting for 98.4% of the haplotype, while the missing BUSCOs were only 0.9% (Table 6). This indicates a relatively complete assembly. We used Merqury v1.3^57^ to estimate the consensus and completeness of the genome assembly. Our results gave a consensus quality value (QV) of 73.38 for the genome assembly, and the completeness value was 99.19% (Table 6). We also used KAT (K-mer Analysis Toolkit) v2.4.0^58^ to estimate the quality of the genome assembly by comparing *k*-mers in HiFi reads and in the assembly. Our results show high consistency between the reads and the genome assembly (Fig. 4a), with each haplotype representing approximately half of the heterozygous peak and nearly all of the homozygous peak (Fig. 4b,c).

**Table 6:** Evaluation of *M. paniculata* genome assembly.

Program	Library	Haplotype A	Haplotype B	Genome
BUSCO	Complete BUSCOs (C)	1,591/98.6%	1,588/98.4%	1,591/98.6%
	Complete and single-copy BUSCOs (S)	1,561/96.7%	1,560/96.7%	9/0.6%
	Complete and duplicated BUSCOs (D)	30/1.9%	28/1.7%	1,582/98.0%
	Fragmented BUSCOs (F)	11/0.7%	12/0.7%	11/0.7%
	Missing BUSCOs (M)	12/0.7%	14/0.9%	12/0.7%
	Total BUSCO groups searched	1,614	1,614	1,614
Merqury	Consensus quality value (QV)	—	—	73.38
	Completeness	—	—	99.19%

In addition, we used BUSCO to evaluate the completeness of the genome annotation by retaining only the longest protein sequence for each gene, and found that the annotation of haplotype A was 97.6% complete, with only 17 (1.1%) genes missing, and the annotation of haplotype B was 97.1% complete, with only 19 (1.2%) genes missing (Table 7), indicating that the annotation was of high quality.

**Table 7:** BUSCO evaluation of *M. paniculata* genome annotation.

Library	Haplotype A	Haplotype B	Genome
Complete BUSCOs (C)	1,576/97.6%	1,567/97.1%	1,591/98.5%
Complete and single-copy BUSCOs (S)	1,553/96.2%	1,541/95.5%	75/4.6%
Complete and duplicated BUSCOs (D)	23/1.4%	26/1.6%	1,516/93.9%
Fragmented BUSCOs (F)	21/1.3%	28/1.7%	9/0.6%
Missing BUSCOs (M)	17/1.1%	19/1.2%	14/0.9%
Total BUSCO groups searched	1,614	1,614	1,614

**Fig. 5:**
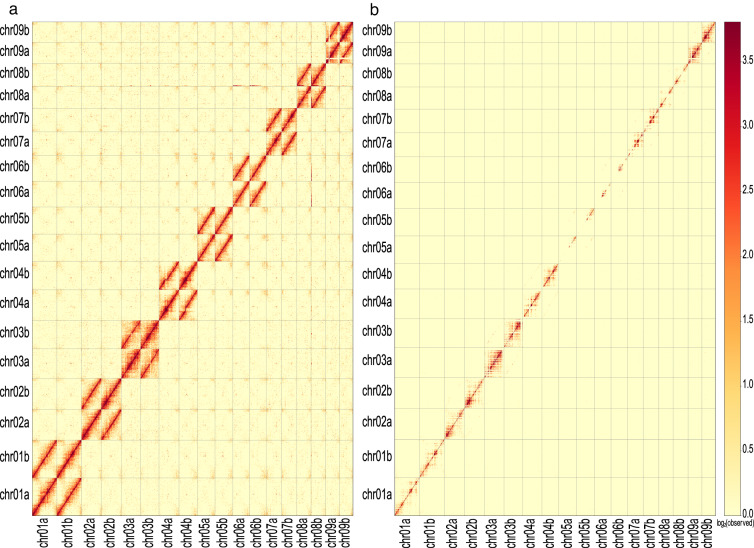
Hi-C interaction heatmap of haplotype A and haplotype B with reads mapping quality ≥0 (including duplicated reads) (**a**) and mapping quality ≥1 (excluding duplicated reads) (**b**). The colour bar indicates the strength of the interaction, with yellow representing low and red representing high.

The Hi-C reads were aligned to the genome assembly using Juicer v1.5.6^10^ with the default parameters. The Juicebox^12^ tools pre command (pre -n -q 0 or 1) was used to convert the raw file generated by Juicer into hic format, and dump command (dump observed BP 100000) was used to extract 100-kb contact matrix from the hic file. The hic file was visualized by Juicebox. Strong interactive signals were observed around the diagonal of the pseudo-chromosomes, and there was no obvious noise outside the diagonal (Fig. 5a), indicating the high quality of this chromosome assembly. In addition, no anomalies were observed across each homologous chromosome pair when duplicated reads were excluded (Fig. 5b), suggesting no switch errors between phased haplotypes.

### Supplementary information

Read coverage depth distribution (50-kb window size, 25-kb window step) along each phased chromosome in both haplotypes

The annotated genes putatively involved in the biosynthesis or metabolism of flavonoids, alkaloids and triterpenoids in Buzhaye

## Data Availability

All commands and pipelines used were performed according to the manuals or protocols of the tools used in this study. The software and tools used are publicly accessible, with the version and parameters specified in the Methods section. If no detailed parameters were mentioned, default parameters were used. No custom code was used in this study.
