# Supplementary material for: Haplotype-resolved chromosomal-level genome assembly of Buzhaye (Microcos paniculata)
Source: Sci Data. 2023 Dec 15;10:901. doi: 10.1038/s41597-023-02821-9 (PMC10724166; doi:10.1038/s41597-023-02821-9)
Supplement: Supplementary file 1 — Read coverage depth distribution (50-kb window size, 25-kb window step) along each phased chromosome in both haplotypes [file 41597_2023_2821_MOESM1_ESM.docx]

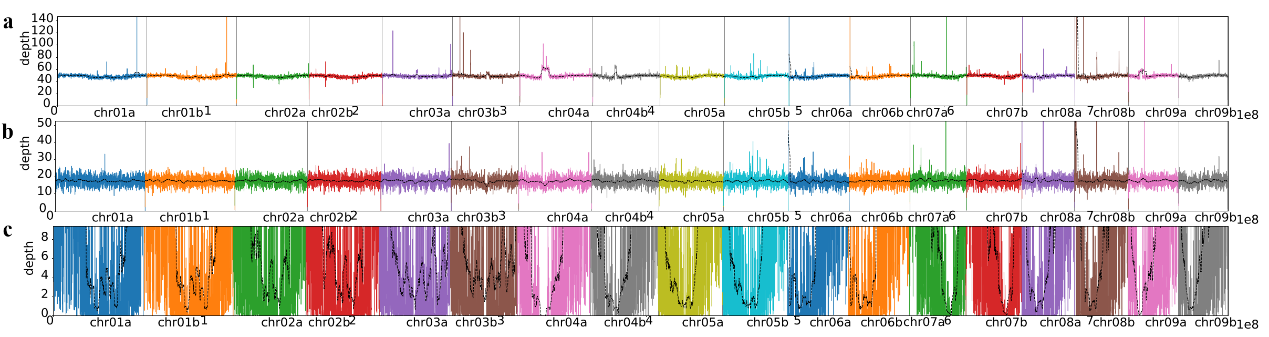


**Figure S1** Read coverage depth distribution (50-kb window size, 25-kb window step) along each phased chromosome in both haplotypes. (a) short read data; (b) long read data; (c) iso-seq data.
